# Molecular mechanisms underlying the renal protective effects of coenzyme Q10 in acute kidney injury

**DOI:** 10.1186/s11658-022-00361-5

**Published:** 2022-07-22

**Authors:** Shankun Zhao, Weizhou Wu, Jian Liao, Xinsheng Zhang, Maolei Shen, Xin Li, Qi Lin, Chaoliang Cao

**Affiliations:** 1 Department of Urology, Taizhou Central Hospital (Taizhou University Hospital), Taizhou, 318000 Zhejiang China; 2 Department of Urology, Maoming People’s Hospital, Maoming, 525000 Guangdong China; 3 Department of Nephrology, Jiaxing Hospital of Traditional Chinese Medicine, Jiaxing, 314001 Zhejiang China; 4 Department of Emergency Medicine, Taizhou Central Hospital (Taizhou University Hospital), Taizhou, 318000 Zhejiang Province China

**Keywords:** Coenzyme Q10, Acute kidney injury, Protection, Mechanism, Antioxidant

## Abstract

Coenzyme Q10 (CoQ10), an endogenous antioxidant, has been reported frequently to exert an outstanding protective effect on multiple organ injury, including acute kidney injury (AKI). In this study, we aim to summarize all the current evidence of the protective action of CoQ10 against AKI as there are presently no relevant reviews in the literature. After a systematic search, 20 eligible studies, either clinical trials or experimental studies, were included and further reviewed. CoQ10 treatment exhibited a potent renal protective effect on various types of AKI, such as AKI induced by drugs (e.g., ochratoxin A, cisplatin, gentamicin, L-NAME, and nonsteroidal anti-inflammatory drug), extracorporeal shock wave lithotripsy (ESWL), sepsis, contrast media, and ischemia–reperfusion injury. The renal protective role of CoQ10 against AKI might be mediated by the antiperoxidative, anti-apoptotic, and anti-inflammatory potential of CoQ10. The molecular mechanisms for the protective effects of CoQ10 might be attributed to the regulation of multiple essential genes (e.g., caspase-3, p53, and PON1) and signaling cascades (e.g., Nrf2/HO-1 pathway). This review highlights that CoQ10 may be a potential strategy in the treatment of AKI.

## Introduction

Acute kidney injury (AKI), a serious kidney disease characterized by a sharp decline in renal function, is one of the common causes of death worldwide [1]. AKI can be induced by various etiologies and pathophysiological processes, and effective treatments are still lacking, particularly in critically ill patients [2]. AKI is not only associated with acute morbidity and mortality, but also with the long-term prognosis of the sufferers and the development of chronic kidney disease, nonrecovery of kidney function, and accelerated progression to end-stage renal disease [3]. It has been reported that up to 50% of patients with AKI are admitted to intensive care units (ICUs) [4, 5]. AKI is associated with poor clinical outcomes, and the mortality rates rise with increasing AKI severity. Patients with serious AKI frequently have worse renal function at the time of hospital discharge [4]. The common risk factors for AKI include sepsis, cardiogenic shock, acute heart failure, surgeries (i.e., cardiac, abdominal, and organ transplantation), trauma, nephrotoxic medications, contrast agents, and chronic kidney disease [6–10]. At present, hemodynamic and fluid status optimization and avoidance of nephrotoxins are the principal therapeutic approaches [9]. However, specific pharmacologic therapies are hampered by late diagnosis, heterogeneous syndromes, variable clinical presentation, and complex pathophysiology, especially in high-risk situations [11]. As a result, there is an urgent need to identify potential therapeutic targets for AKI [12].

Coenzyme Q10 (CoQ10), a fat-soluble lipophilic molecule ubiquitously located at the hydrophobic domain of cell membranes, is an endogenous antioxidant that is partially involved in the process of energy metabolism and antioxidant protection [13, 14]. CoQ10 serves as an electron and proton carrier of the mitochondrial respiratory chain, playing an essential role in ATP synthesis by enabling the process of oxidative phosphorylation [15, 16]. CoQ10 controls cell redox status and regulates reactive oxygen species (*ROS*) generation, exhibiting its effects on protecting the cell against free-radical-induced oxidation [17]. In addition, CoQ10 also has anti-inflammatory action, with capability to inhibit inflammatory gene expression [18, 19]. Moreover, CoQ10 may play an important role in the immune system by regulating lysosomal and peroxisomal function during the immune response [20]. On the basis of these unique properties of CoQ10, both clinical trials and experimental studies described that CoQ10 supplementation had an outstanding protective effect on acute organ injuries (i.e., cerebral, myocardial, lung, liver, and renal injury) in recent years [21–25].

Accumulating evidence demonstrates that CoQ10 plays a crucial role in protecting AKI, which might be attributed to the functions of CoQ10, including anti-inflammatory effects, gene expression regulation, enhancement of the activity of antioxidant enzymes, free-radical scavenging, and lipid bilayer membrane stabilization [13, 25, 26]. Currently, there are no narrative reviews addressing the roles of CoQ10 in AKI. We present a first attempt to summarize all the evidence on the proposed roles of CoQ10 against AKI via a comprehensive review. The objective of this study is to provide readers with an overview of the current status of this topic that may facilitate the clinical application of CoQ10 in treating AKI.

### Overview of CoQ10

CoQ10, a fat-soluble organic molecule similar to a vitamin, is endogenously synthesized by human cells [27]. According to PubChem, it comprises a benzoquinone group and a poly-isoprenoid side chain of ten isoprenoid units in humans (https://pubchem.ncbi.nlm.nih.gov/compound/5281915). Its molecular formula is C_59_H_90_O_4_ (molecular weight 863.3 g/mol), also known as ubidecarenone, coenzyme Q10, and ubiquinone-10. CoQ10 can be absorbed from the small intestine into the lymphatic system and then enter the blood circulation; bile is the main elimination route [28]. Higher amounts of CoQ10 are observed in tissues with high energy requirements or metabolic activity, e.g., heart, kidney, liver, and muscle [29]. The following pharmacokinetic properties of CoQ10 have been reported: area under the curve of 11.51 μg h/ml and *C*_max_ of 0.32 μg/ml at a time of 7.9 h (https://pubchem.ncbi.nlm.nih.gov/compound/5281915, Section: 8.4 Absorption, Distribution and Excretion). The half-life of CoQ10 is reported to be 21.7 h (https://pubchem.ncbi.nlm.nih.gov/compound/5281915, Section: 8.6 Biological Half-Life). CoQ10 can be metabolized in all tissues by phosphorylation in the cells and then transportation to the kidneys. CoQ10 is frequently found in cell membranes, particularly in mitochondria [30]. CoQ10 exerts its biological effect largely on the basis of its lipophilic antioxidant capacity, scavenging free radicals by suppressing the initiation and development of lipid peroxidation in cell membranes [31]. CoQ10 appears generally in two forms: reduced (ubiquinol) and oxidized (ubiquinone) [32]. Both coexist and regenerate each other via sequential redox reactions (Q cycle) [33]. Ubiquinol shows antioxidant behaviors in cell and organelle membranes, reducing oxidative stress and lipid peroxidation and regenerating vitamins C and E back to their active form [29]. Meanwhile, ubiquinone commonly acts as an excellent electron carrier in the mitochondrial electron transport chain in most eukaryotes [34]. The levels of CoQ10 are high in human organs with high metabolic activity (e.g., liver, kidney, and heart) [35]. CoQ10 is involved in the production of adenosine triphosphate (ATP), regulating mitochondrial respiratory chain complexes. CoQ10 also participates in many human or rodent physiological processes, including sulfide oxidation, regulation of mitochondrial permeability transition pore, and translocation of protons and calcium ions across biological membranes [14, 36]. CoQ10 deficiencies have been found in patients with various diseases, including cancers, cardiovascular diseases (e.g., statin myopathy, congestive heart failure, and hypertension), diabetes mellitus, dementia, hepatitis, Parkinson’s disease, skin aging, and renal diseases [37–40].

### Literature search

**Fig. 1:**
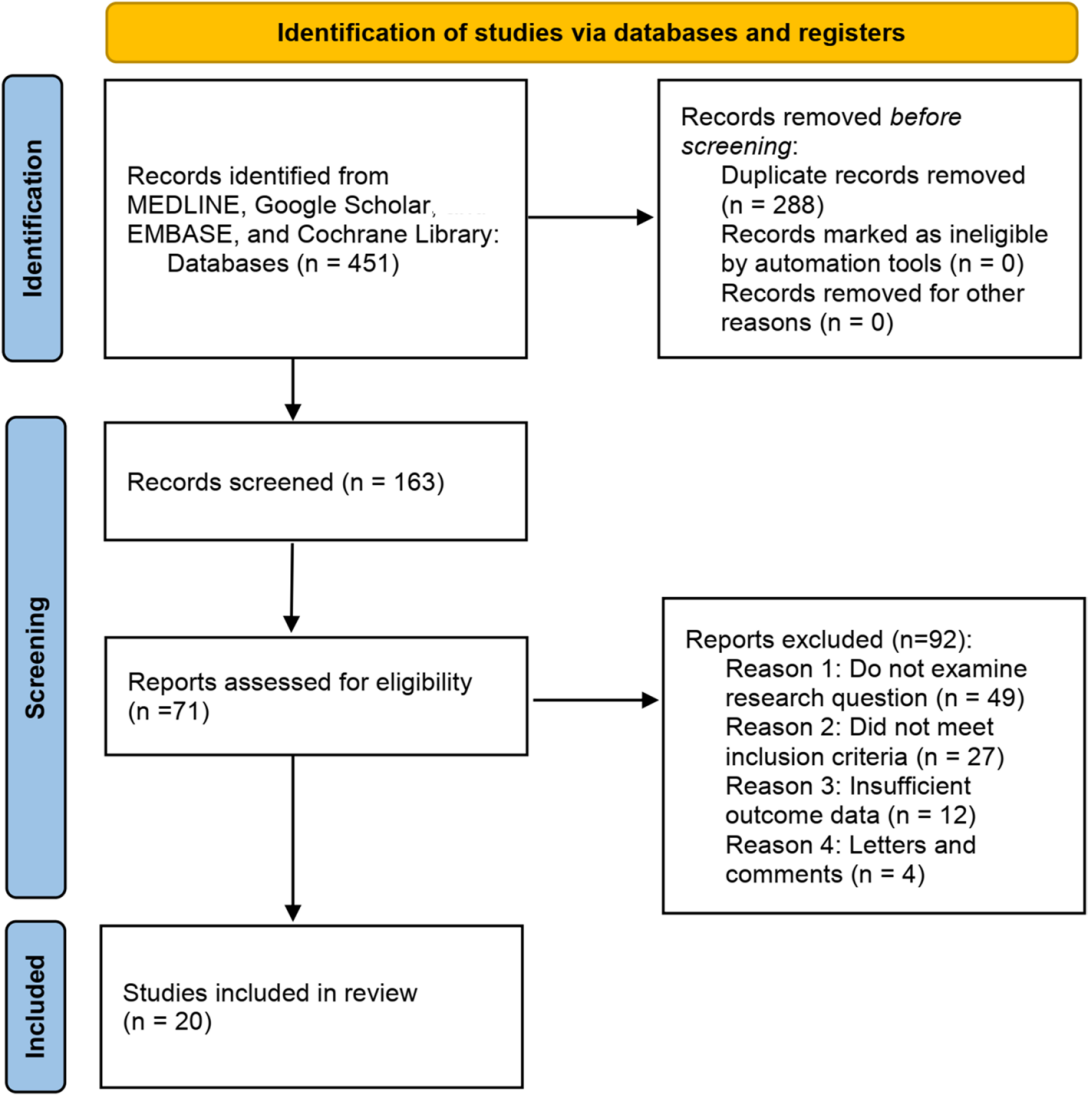
Search flowchart for identifying the relevant studies reporting use of CoQ10 to treat AKI

To maximally identify the eligible studies relevant to our topic of CoQ10 in treating or preventing AKI, we performed a systematic review of the most commonly used databases, i.e., MEDLINE, Google Scholar, EMBASE, and Cochrane Library. The keyword search strategy in MEDLINE was: ((((((((((((((((“coenzyme Q10” [Supplementary Concept]) OR (2,3-dimethoxy-5-methyl-6-decaprenylbenzoquinone)) OR (CoQ 10)) OR (co-enzyme Q10)) OR (ubidecarenone)) OR (ubiquinone 50)) OR (ubiquinone Q10)) OR (Bio-Quinone Q10)) OR (ubiquinone 10)) OR (CoQ10)) OR (ubisemiquinone radical)) OR (Q-ter)) OR (ubisemiquinone)) OR (coenzyme Q10, (Z,Z,Z,Z,Z,Z,E,E,E)-isomer)) OR (coenzyme Q10, ion (1-), (all-E)-isomer)) OR (Q10)) AND ((((Kidney Failure) OR (Renal Failure)) OR (Kidney injury)) OR (renal injury)). Figure 1 shows the search flowchart for screening the relevant studies. The inclusion criteria for study eligibility include the following: (1) clinical study reporting on the effects of CoQ10 in treating patients with AKI; (2) experimental research reporting on the roles of CoQ10 in AKI and its potential molecular mechanisms; (3) any clinical or experimental studies reporting on the effects of ubiquinol and ubiquinone in AKI. Twenty studies [25, 26, 41–58], either clinical or experimental, were finally included for further analysis and summary. A specific data collection table was used to extract the main data from each study, including article information (e.g., the first author’s name, publication year), research object (e.g., cell/animal model or patient), types of AKI, CoQ10 administration, associated genes/pathways and agents, and the main findings of the study. Table 1 presents a summary of the relevant studies reporting CoQ10 against AKI.

**Table 1:** Characteristics and main findings of the 20 eligible studies reporting use of CoQ10 to treat AKI

Study/references	Research object	Types of injury	CoQ10 administration	Associated genes/pathways and agents	Main findings
Yenilmez et al. 2010 [25]	Rats	Induced by ochratoxin A (2.2 mg/kg, gastric gavage)	10 mg/kg, intraperitoneally	NA	CoQ10 treatment ameliorated the ochratoxin A-induced renal oxidative injuries
Fouad et al. 2010 [41]	Mice	Acute cisplatin (5 mg/kg, i.p.) nephrotoxicity injury	10 mg/kg, intraperitoneally	Downregulating* iNOS*, *NF-κB,* *caspase-3*, and *p53*	CoQ10 protects against acute cisplatin nephrotoxicity by decreasing the expression of iNOS, NF-κB, caspase-3, and p53 in renal tissue
Ahmadvand et al. 2014 [26]	Rats	Gentamicin-induced nephrotoxicity injury	15 mg/kg, intraperitoneally	Downregulating *PON1*	CoQ10 alleviated gentamicin-induced nephrotoxicity by reducing the elevated serum lipid peroxidation, lipid profile and atherogenic index, and *PON1* activity
Carrasco et al. 2014 [42]	Patients (*n* = 100)	ESWL-induced kidney injury	200 mg/day, orally administered during the week before ESWL and for 1 week after	Clinical trial	Compared with placebo group, CoQ10 significantly increased glomerular filtration (*P* = 0.013) and decreased albumin/creatinine and β2-microglobulin level (*P* = 0.02)
Fatima et al. 2015 [43]	Rats	Cisplatin-induced oxidative stress injury	10 mg/kg, intraperitoneally	CoQ10 combined with EGCG was more effective in attenuating renal injury	CoQ10 was effective against cisplatin-induced nephrotoxicity, resulted in a significant reduction of BUN and serum creatinine level
Fatima et al. 2016 [44]	Rats	Cisplatin-induced nephrotoxicity injury (7 mg/kg, i.p.)	5 mg/kg, intraperitoneally	Combined with 15 mg/kg EGCG	Combined CoQ10 and EGCG significantly attenuated cisplatin-induced oxidative stress, nitrosative stress, and inflammatory and apoptotic parameters
Ozer et al. 2017 [45]	Rats	Cecal ligation and puncture-induced sepsis	10 mg/kg, intraperitoneally	NA	CoQ10 showed protective effects against sepsis-induced kidney injury by anti-inflammatory and antioxidative effects
Arany et al. 2017 [46]	Renal proximal tubule cell line	Nicotine-induced renal cell injury (cells treated with 200 µM nicotine)	10 µM	Serine 36 phosphorylation	CoQ10 significantly inhibited nicotine-mediated production of reactive oxygen species (*ROS*) and consequent apoptosis
Ustuner et al. 2017 [47]	Rats	Gentamicin-induced kidney damage (80 mg/kg/day, i.p.)	10 mg/kg, intraperitoneally	NA	Necrotic tubuli rate and hyalin accumulation in tubuli were decreased after CoQ10 treatment
Shamardl et al. 2017 [48]	Rats	L-NAME hypertensive kidney injury (40 mg/kg, i.p.)	10 mg/kg, intraperitoneally	Combination with vitamin D had further effects on all parameters	CoQ10 decreased systolic, diastolic, and mean arterial pressure, total cholesterol, LDL-C, creatinine, TNF-α, and malondialdehyde level
Chen et al. 2018 [49]	Patients (*n* = 150), rats (*n* = 45)	Contrast-induced nephropathy	Patients: 20 mg three times daily from 2 days before to 3 days after procedure; rats: 20 mg/kg	Combined with 20 mg trimetazidine	Incidence of contrast-induced nephropathy was significantly lower in CoQ10 plus trimetazidine group compared with control group (6.67% versus 21.3%, *P* = 0.01); CoQ10 and trimetazidine significantly reduced oxidation stress in an AKI animal model
Akbulut et al. 2019 [50]	Rats	Renal ischemia–reperfusion injury	10 mg/kg, intraperitoneally	NA	CoQ10 decreased tissue oxidative stress levels and scores of histopathology and apoptosis
Albadrany et al. 2019 [51]	Broiler chickens	Diclofenac-induced renal injury (1 and 2 mg/kg, i.p.)	30 mg/kg, orally	NA	CoQ10 could not alleviate diclofenac-induced renal injury, but worsened impaired renal function
Kennedy et al. 2020 [52]	Mice	Khat-induced nephrotoxicity (1500 mg/kg, gastric gavage)	200 mg/kg, orally	Normalization of *GSH* and *TNF-α* expression	CoQ10 decreased creatinine levels and reduced tubular necrosis and tubular epithelium injury
Megrin et al. 2020 [53]	Rats	Lead-acetate-induced renal injury	10 mg/kg, intraperitoneally	Upregulation *Nrf2/HO-1* pathway	CoQ10 reduced the deleterious cellular side effects of lead acetate exposure owing to its antioxidant, anti-inflammatory, and anti-apoptotic effects
Abdeen et al. 2020 [54]	Rats	Piroxicam-induced oxidative injury	10 mg/kg, orally	NA	CoQ10 attenuated the piroxicam-inflicted deleterious oxidative harm and apoptosis, improving mitochondrial function and reducing ROS, which might be ascribed to the free-radical scavenging activity of CoQ10
Liu et al. 2020 [55]	Mice	Renal ischemia–reperfusion injury	50 mg/kg, NA	NA	CoQ10 reduced oxidative damage in vitro and in vivo, inhibited renal cell apoptosis, and attenuated inflammatory response in renal I/R injury model, thus improving renal function
Liu et al. 2021 [56]	Mice	Renal ischemia–reperfusion injury	50 mg/kg, tail vein injection	NA	The mitochondria-targeted triphenylphosphine CoQ10 nanoparticles alleviated mtDNA damage, suppressed inflammatory and apoptotic responses, and improved renal function
Alshogran et al. 2021 [57]	Rats	Contrast-induced kidney injury	20 mg/kg, orally	Combined with 10 mg/kg atorvastatin	Pretreatment with CoQ10/atorvastatin showed regenerative effect on distal tubules with mild kidney histology alterations as compared with contrast-induced nephropathy rats
Couto et al. 2021 [58]	Rats	Contrast-induced acute kidney injury	10 mg/kg, intraperitoneally	NA	CoQ10 ameliorated renal function, prevented hemodynamic changes, neutralized oxidative damage, and prevented the progression of histologic damage

*CoQ10* coenzyme Q10, *AKI* acute kidney injury, *iNOS* inducible nitric oxide synthase, *NF-κB* nuclear factor-κB, *PON1* paraoxonase 1, *HO-1* heme oxygenase 1, *ESWL* extracorporeal shock wave lithotripsy, *EGCG* epigallocatechin gallate, *MnSOD* manganese superoxide dismutase, *Nrf2* nuclear factor erythroid 2-related factor 2, *L-NAME* l-arginine analog, *BUN* blood urea nitrogen, *SCR* serum creatinine, *ROS* reactive oxygen species, *i.p.* intraperitoneally

Among the 20 included studies in Table 1, only 2 clinical trials were available and both of them suggested that administration of CoQ10 significantly improved renal function in patients with AKI. The AKI was either ESWL- or contrast-induced. In addition to one clinical study, there were two animal studies reporting renal protective effects against contrast-induced AKI, while only one clinical trial reported CoQ10 as preventing ESWL-associated AKI. Although three experimental studies reported the role of CoQ10 in renal ischemia–reperfusion injury, no relevant human trial is currently available. Therefore, contrast-induced AKI might be the type of AKI most successfully treated with CoQ10.

### Clinical implications of CoQ10 in AKI

Among the 20 included studies, two clinical trials reported on the clinical significance of CoQ10 in AKI. Carrasco et al. [42] conducted a prospective, randomized, double-blind, placebo-controlled clinical trial of 100 patients with AKI induced by extracorporeal shock wave lithotripsy (ESWL). These patients were divided randomly into two groups administered either CoQ10 (200 mg/day) or placebo (orally) during the week before ESWL and for 1 week after. The results showed that CoQ10 significantly increased glomerular filtration (*P* = 0.013) and decreased albumin/creatinine and β2-microglobulin levels (*P* = 0.02) compared with the placebo group. Furthermore, CoQ10 was also associated with an improvement in vasoactive hormone parameters (e.g., renin and aldosterone), vascular resistance index, and interleukin levels. Of note, CoQ10 administration did not significantly affect the parameters of oxidative stress (e.g., *LPO, SOD, GPx, and GSH*). Carrasco et al.’s study revealed that CoQ10-treated peri-ESWL dramatically improved renal function of ESWL-induced AKI; meanwhile, it also strengthened the vasoactive and inflammation parameters. A more recent randomized, paralleled-arm, double-blind, controlled trial conducted by Chen et al. demonstrated that 21 (14.00%) of the 150 patients developed contrast-induced AKI undergoing elective cardiac catheterization. The authors found that patients administered 20 mg CoQ10 plus trimetazidine (TMZ) three times daily from 2 days before to 3 days after the procedure had a significantly lower rate of contrast-induced AKI as compared with the placebo group (6.67% versus 21.3%, *P* = 0.01). On multiple logistic regression analysis, CoQ10 plus TMZ was an independent protective factor against contrast-induced AKI [odds ratio (OR) 0.252, 95% confidence interval (CI) 0.082–0.774, *P* = 0.016]. This study further suggested that CoQ10 and TMZ significantly reduced the concentration of BUN and SCR, oxidation stress, and tubular pathological injuries. Taken together, the above two clinical studies revealed that CoQ10 might serve as a crucial protective drug in preventing AKI of either intrarenal (ESWL) or extrarenal cause (contrast-induced nephropathy).

## Protective properties of CoQ10 in AKI reported in experimental studies

### Roles of CoQ10 in drug- or substance-induced AKI

Drug-induced nephrotoxicity is one of the most common causes of AKI, accounting for approximately 20% of all community- and hospital-acquired events [59]. In addition, drug-induced nephrotoxicity is the main reason for the failures of some phase III clinical trials [60]. A better understanding of the potential mechanisms underlying drug-induced AKI is gradually achieved. More importantly, there have been significant advances in AKI therapy in recent years. CoQ10 is among the most intensely investigated therapies in AKI treatment.

**Fig. 2:**
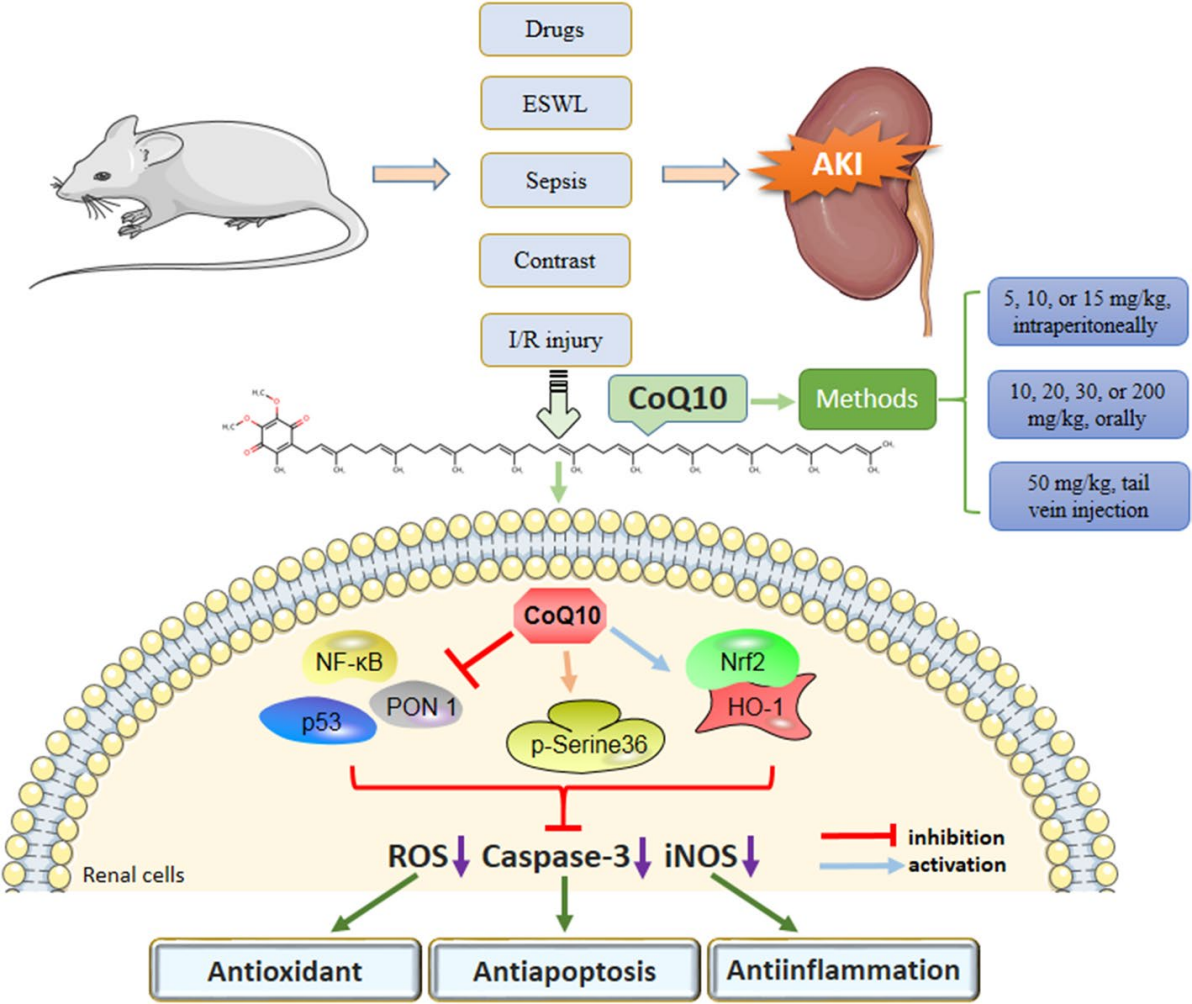
Main molecular mechanisms of the renal protective effects developed by CoQ10 in different types of AKI. CoQ10 is an endogenous antioxidant. Under AKI treatment with CoQ10, multiple associated genes (e.g.,* iNOS, caspase-3, NF-κB, p53*, and *PON1*) and a series of downstream signaling pathways (e.g., *Nrf2/HO-1* pathway) were regulated, resulting in antioxidant, anti-apoptotic, and anti-inflammatory effects. *CoQ10* coenzyme Q10, *AKI* acute kidney injury, *ESWL* extracorporeal shock wave lithotripsy, *iNOS* inducible nitric oxide synthase, *PON1* paraoxonase 1, *HO-1* heme oxygenase 1, *ROS* reactive oxygen species, *Nrf2* nuclear factor erythroid 2-related factor 2

Figure 2 illustrates the potential molecular mechanisms of the renal protective effects of CoQ10 against AKI.

### Ochratoxin A-associated AKI

Ochratoxin A (OTA), one of the secondary metabolites of fungi (e.g., *Aspergillus niger* and *Aspergillus ochraceus*), seriously impairs human health through its cytotoxicity and embryotoxicity [61]. OTA exhibits nephrotoxic effects by inducing tubulointerstitial nephritis. The underlying mechanism of OTA nephrotoxicity may be associated with the repression of protein synthesis, genotoxic impacts, and oxidative damage. Yenilmez et al. [25] established an OTA-induced AKI rat model and found that 10 mg/kg CoQ10 remarkably ameliorated OTA-induced renal oxidative injuries (e.g., ROS damage), accompanied by increasing glutathione and decreasing malondialdehyde levels in the plasma. This experimental study implies that CoQ10 might be a potential therapeutic measure for preventing OTA-induced AKI in clinical practice.

### Cisplatin-induced AKI

Chemotherapy is a conventional mode of treatment for multiple cancers [62]. Cisplatin is one of the most commonly used chemotherapeutic agents for treating various solid tumors, including breast, ovarian, testicular, head, and neck cancers [63, 64]. Despite its antineoplastic efficacy, however, treatment with cisplatin frequently causes toxicity-related symptoms, such as dose-related nephrotoxicity-induced AKI. It was reported that AKI commonly occurred after an initial dose of cisplatin [65]. Among multitudinous mechanisms in the action of cisplatin-associated AKI, oxidative stress with increased generation of reactive oxygen species may be a key etiological factor. Several antioxidants have been confirmed to exert a protective effect against the nephrotoxicity of cisplatin [66]. A previous study demonstrated that CoQ10 treatment (10 mg/kg/day, intraperitoneally) dramatically ameliorated cisplatin-induced AKI in mice [41]. The molecular mechanisms underlying this potential were speculated to be the downregulation of *iNOS, NF-κB, caspase-3*, and *p53* in renal tissue, thus protecting against renal cell apoptosis. In line with this study, Fatima et al. [43] also found that CoQ10 (10 mg/kg, intraperitoneally) played a crucial role in protecting cisplatin-induced AKI. In addition, the authors suggested that CoQ10 combined with epigallocatechin gallate (EGCG) was more effective at attenuating renal injury. CoQ10 exerts its protective effect by decreasing BUN and serum creatinine levels. Besides, CoQ10 significantly attenuated cisplatin-induced alterations in the renal tissue concentration of Se, Zn, and Cu ions compared with the control group. Furthermore, another study by Fatima et al. revealed that 5 mg/kg CoQ10 combined with 15 mg/kg EGCG substantially alleviated cisplatin-induced oxidative stress, nitrosative stress, and inflammatory and apoptotic parameters in a rat model. The above three studies highlight that CoQ10 might serve as a promising therapeutic option to protect against cisplatin-mediated AKI encountered in clinical practice.

### Gentamicin-induced AKI

Gentamicin is a well-known aminoglycoside antibiotic that is applied against the majority of Gram-negative microorganism infections [67]. However, approximately 30% of patients under the treatment of aminoglycosides for over 7 days present with some symptoms of nephrotoxicity [68], which may be associated with the development of apoptosis and necrosis as well as the production of oxidative stress. Gentamicin-induced AKI or nephrotoxicity is commonly represented by a high level of urea and creatinine with tubular necrosis [69]. Paraoxonase 1 (*PON1*), an antioxidant enzyme, effectively protects LDL and HDL from oxidation and plays role in atherosclerosis prevention. Ahmadvand et al. [26] reported that CoQ10 (15 mg/kg, intraperitoneally) markedly alleviated gentamicin-induced AKI by reducing the elevated serum lipid peroxidation and PON1 activity. Inconsistent with Ahmadvand et al.’s study, a subsequent experimental study showed that CoQ10 did not significantly alter the nephrotoxicity parameters [47]. However, the necrotic tubuli rate and hyalin accumulation in tubuli were decreased after CoQ10 treatment. The authors concluded that CoQ10 administration provided a protective effect on the kidney against gentamicin-induced AKI by the antioxidant and anti-inflammatory properties of CoQ10 [47]. Though widely used in clinical practice, gentamicin is a nephrotoxic antibiotic. However, as some specific pathogens are sensitive to gentamicin, it is definitely not negligible. On the basis of the above in vivo studies, CoQ10 shows renal protective effects on gentamicin-induced AKI.

### Nicotine-induced AKI

High levels of nicotine, one of the main constituents of tobacco smoke, are observed in the kidneys of chronic smokers. Nicotine has been found to cause apoptosis in renal proximal tubule cells by elevating the production of reactive oxygen species [70]. It has been suggested that chronic nicotine exposure could also induce AKI [71]. An in vitro study conducted by Arany et al. [46] demonstrated that treatment with 10 µM CoQ10 significantly repressed nicotine-mediated production of reactive oxygen species and consequent apoptosis in a nicotine-induced acute renal proximal tubule cell injury model. The renal protective properties of CoQ10 administration might be correlated with enhancement of p66shc promoter, phosphorylation of serine 36, and activation of Nrf2/MnSOD, thus counterbalancing ROS expression and anti-apoptosis. Clinically, if AKI is unequivocally caused by nicotine, CoQ10 provides a clinical benefit to these sufferers.

### L-NAME hypertensive AKI

*N*-nitro-l-arginine methyl ester (L-NAME) is a nitric oxide synthase inhibitor. It is commonly used to establish hypertensive animal models by causing nitric oxide (NO) deficiency and therefore increasing total peripheral resistance and blood pressure [72]. It is known that NO can be synthesized and released from endothelial cells, thus inducing vasorelaxation. L-NAME causes hypertension by reducing NO. Also, L-NAME is confirmed to elevate oxidative stress in an animal model. Shamardl et al. [48] reported that CoQ10 (10 mg/kg, intraperitoneally) drastically decreased the systolic, diastolic, and mean arterial pressure, total cholesterol, LDL-C, creatinine, *TNF-α*, and malondialdehyde levels. CoQ10 was also found to elevate the total antioxidant capacity in kidney tissue. Belardinelli et al. [73] suggested that the protective effect of CoQ10 on the cardiovascular system might be correlated with its potent chain-breaking lipid-soluble antioxidant effects, counteracting vasoconstriction and preventing oxidative stress and inflammation by recoupling of nitric oxide synthase. Besides, CoQ10 exhibits the inhibitory effects of lipoprotein α receptors and the dietary lipid absorption of cholesterol [74, 75]. Shamardl et al. [48] suggested that the renal protective mechanisms of CoQ10 might be due to the effects of CoQ10 serving as an antioxidant, anticytokine, and blood pressure conserver. Furthermore, the authors indicated that the combination of CoQ10 and vitamin D had further effects on all parameters.

### NSAID-induced AKI

Piroxicam is a common nonsteroidal anti-inflammatory drug (NSAID) that belongs to the oxicam class. Piroxicam is frequently prescribed for several painful and inflammatory events, e.g., postoperative, rheumatoid arthritis, and even cancer pain [76]. However, piroxicam can induce hepatorenal damage due to oxidative stress and disruption of cellular redox homeostasis. Diclofenac metabolites can lead to the apoptosis of hepatocytes and cause mitochondrial malfunction, resulting in liver damage. Besides, diclofenac also has a detrimental effect on the kidneys by inhibiting prostaglandin. It was reported that diclofenac can mediate nephrotoxicity, causing elevation of urea, creatinine, and electrolytes (e.g., Na, K, and Cl) [77]. Abdeen et al. [54] showed that CoQ10 (10 mg/kg, orally) significantly attenuated piroxicam-inflicted deleterious oxidative harm and apoptosis in an AKI model, improving mitochondrial function and reducing ROS in kidney tissue. The renoprotective action of CoQ10 might be attributed to its free-radical scavenging activity.

Diclofenac is an NSAID commonly used in the field of veterinary medicine. Diclofenac has the function of alleviating pain and has anti-inflammatory and antipyretic effects [78]. However, diclofenac is also nephrotoxic, causing severe necrosis of cells lining renal tubules. Albadrany et al. [51] demonstrated that CoQ10 could not alleviate diclofenac-induced renal injury, but worsened the impaired renal function in a broiler chicken model. Obviously, this study exhibits a quite opposite trend of the roles of CoQ10 in AKI. It was suggested that COQ10 co-administered with diclofenac might cause a synergistic detrimental effect on renal tissue [51]. Moreover, it was speculated that COQ10 might inhibit prostaglandin synthesis in the kidney, similar to the biological effects of NSAIDs [51]. Moreover, CoQ10 could also repress the formation of NO, resulting in overcontraction of blood vessels [79]. On the basis of these theories, CoQ10 might impair renal function. Unlike for the above types of AKI, CoQ10 is not recommended to prevent or treat NSAID-induced AKI, as CoQ10 can aggravate impaired renal function.

### Khat-induced AKI

Khat (*Catha edulis*, Forsk) is an evergreen shrub used for recreational purposes. Owing to its psychostimulant effects, the consumption of khat causes substance abuse in some countries. Several alkaloids, including cathinone, cathine, and norephedrine, are accountable for the effects of khat. Khat has a negative effect on the physiological and biochemical processes of the kidney, causing nephrotoxicity [80]. Since CoQ10 has been shown to have potent antioxidant and anti-inflammatory effects, Kennedy et al. [52] investigated the exact roles of CoQ10 in khat-induced AKI and found that CoQ10 (200 mg/kg, orally) significantly decreased creatinine levels and reduced tubular necrosis and tubular epithelium injury. The protective effect derived from CoQ10 might be associated with the reduction of oxidative stress and inflammation

### Lead-acetate-induced AKI

Lead acetate (AcPb) is a raw material used in chemical industries. The hazardous effects of AcPb are commonly due to the presence of lead (Pb) [81]. Pb can induce oxidative stress and generation of reactive oxygen species in tissues. Exposure to Pb may induce Pb accumulation in the proximal tubules and thus result in renal injury and eventually kidney failure [82]. Megrin et al. [53] discovered that CoQ10 (10 mg/kg, intraperitoneally) reduced the deleterious cellular side effects of AcPb exposure owing to its antioxidant, anti-inflammatory, and anti-apoptotic effects. CoQ10 significantly decreased the level of tumor necrosis factor-α, interleukin-1β, Bax, and *caspase-3* in the kidney. The molecular mechanisms might be related to the upregulation of *Nrf2* and *HO-1* expression.

### Sepsis-associated AKI

Sepsis, a severe systemic inflammatory response related to various infections, remains one of the leading causes of morbidity and mortality in hospitals [83, 84]. Severe sepsis may induce multiorgan dysfunction. The kidney is an organ susceptible to sepsis, especially severe sepsis. Despite intensive treatment strategies, sepsis is life threatening. Ozer et al. revealed that CoQ10 (10 mg/kg, intraperitoneally) exerted protective effects on cecal ligation and puncture-induced sepsis-induced AKI via its anti-inflammatory and antioxidative effects. However, further clinical trials are warranted to confirm this in vivo finding.

### Roles of CoQ10 in contrast-induced AKI

Contrast-induced AKI is an iatrogenic AKI that has long been observed after intravascular administration of contrast medium for angiography and percutaneous coronary interventions [85]. Contrast-induced nephropathy is the third-leading cause of acquired AKI in hospitals. Contrast medium exposure, either intra-arterially or intravenously, may result in AKI. The incidence of AKI after intravenous contrast medium administration has been reported at 5–6% [85]. Contrast-induced AKI may cause higher mortality, greater treatment costs, and prolonged hospitalization. It was reported that contrast medium could induce the apoptosis of renal tubular epithelial cells by abnormally increasing the level of ROS [86]. Since CoQ10 is a strong antioxidant, it may play a crucial role in contrast-induced AKI.

To investigate the effects of CoQ10 in contrast-induced AKI, Chen et al. [49] found that 20 mg/kg CoQ10 treatment dramatically reduced serum BUN and creatinine levels, as well as oxidation levels in kidney tissue. A combination of CoQ10 and trimetazidine significantly decreased the necrosis of tubular epithelial cells and the cast formation. In line with this finding, Alshogran et al. [57] demonstrated that pretreatment with CoQ10 (20 mg/kg, orally) and atorvastatin (10 mg/kg, orally) exhibited regenerative effects on distal tubules with mild kidney histology alterations after contrast-induced AKI. Also, a recent experimental study conducted by Couto et al. suggested that CoQ10 (10 mg/kg, intraperitoneally) significantly ameliorated renal function in an animal model of contrast-induced AKI. CoQ10 administration could prevent hemodynamic changes, neutralize oxidative damage, and alleviate the progression of histologic damage compared with the contrast-induced AKI group. On the basis of the above three relevant studies, CoQ10 could serve as a promising strategy to prevent contrast-induced AKI in clinical practice.

### Ischemia–reperfusion-induced AKI

Renal ischemia–reperfusion injury (RIRI), characterized by restriction of blood supply to the kidney, can cause renal cell death and lead to renal failure [87]. It commonly occurs after organ transplantation, infarction, and sepsis. RIRI is one of the main causes of AKI. RIRI may activate and exacerbate multiple inflammatory responses, thus increasing the production of ROS, chemokines, leukocytes, and cytokines. CoQ10 is a potent antioxidant and free-radical scavenger, with mounting evidence demonstrating that it could protect the kidney from ischemia–reperfusion (I/R) injury.

Akbulut et al. [50] showed that CoQ10 (10 mg/kg, intraperitoneally) could significantly decrease the tissue oxidative stress levels and the scores of histopathology and apoptosis in a RIRI-induced AKI rat model. Consistent with Akbulut et al.’s findings, Liu et al. [55] also found that CoQ10 treatment substantially improved renal function by reducing oxidative damage, inhibiting renal cell apoptosis, and attenuating inflammatory response. However, Liu et al. [56] applied an RIRI-induced AKI mouse model and used a CoQ10 dose of of 50 mg/kg. In a subsequent study developed by Liu et al., the authors further found that mitochondria-targeted triphenylphosphine CoQ10 nanoparticles remarkably alleviated mtDNA damage, suppressed inflammatory and apoptotic responses, and improved renal function in the RIRI animal models. The way of CoQ10 administration was 50 mg/kg via tail vein injection. These studies indicated that CoQ10 effectively protected renal function of RIRI-induced AKI through its antiperoxidative, anti-apoptotic, and anti-inflammatory potential. In the future, CoQ10 may have broad clinical application in the treatment of ischemia–reperfusion-induced AKI.

### Roles of mitochondrial function in the action of CoQ10 against AKI

CoQ10 plays a key role in cellular energy supply through oxidative phosphorylation within mitochondria [88]. Mitochondrial dysfunction might cause oxidative stress, systemic inflammatory responses, and cell apoptosis, thus inducing renal damage. Yenilmez et al. [25] demonstrated that CoQ10 ameliorated ochratoxin-A-induced AKI partially by inhibiting ROS damage in mitochondria. Ozer et al. [45] revealed that CoQ10 deficiency induced by sepsis could cause progressive mitochondrial failure and energy depletion. CoQ10 was found to prevent cell apoptosis by maintaining the mitochondrial permeability transition pore [89]. A recent study showed that CoQ10 protected against lead-acetate-induced AKI in rats by preventing apoptosis [53]. The toxic effects of piroxicam might be associated with mitochondrial dysfunction and excess generation of ROS [90]. Abdeen et al. [54] reported that CoQ10 improved mitochondrial function in piroxicam-inflicted AKI. Liu et al. [56] revealed that mitochondria-targeted T-NP_CoQ10_ nanoparticles were detected to play an important protective role in renal I/R injury by dramatically reducing the oxidative levels and inflammatory responses in the tissues of AKI. On the basis of the above evidence, the protective effects elicited by CoQ10 on AKI might be attributed partially to the recovery of mitochondrial activity and the elevation of energy production in renal cells.

### Directions for future experimental research

As shown in Table 1, most of the included in vivo studies suggest that CoQ10 might have a renal protective role in drug- or substance-induced AKI, although the molecular mechanisms of CoQ20 were multifaceted. The involved genes or pathways were inconsistent among different studies. Since CoQ10 exhibits antioxidant, anti-inflammatory, and anti-apoptotic effects, future experiments could be more concentrated on those associated genes or pathways that regulate the biological functions of CoQ10. For example, *Nrf2* was found to be correlated with both antioxidant and anti-apoptotic effects of CoQ10 in various organ injuries [53, 91–93]. In regard to the anti-inflammatory properties of CoQ10, *NF-ĸB* expression was detected to be one of the key factors in the anti-inflammatory effect of CoQ10 in acute brain injury and other diseases [94, 95]. On the basis of the current evidence, the exact biological functions of *Nrf2* and *NF-ĸB* in the actions of CoQ10 in treating AKI deserve further future investigation. After confirmation, CoQ10 combined with those drugs targeting *Nrf2* and *NF-ĸB* might exert an excellent renal protective effect on AKI.

Another point of concern is that CoQ10 supplementation may causes an increase in renal CoQ10 status. Since repeated biopsies cannot be undertaken, blood CoQ10 analysis may be reliable for monitoring CoQ10 treatment, while urinary CoQ10 analysis can provide information about the quantity of CoQ reaching the target tissues, such as the kidney [96]. Several studies [97, 98] showed that CoQ10 and its binding proteins decreased in renal injury diseases. Therefore, the protective effect exerted by CoQ10 in AKI might be associated with the amelioration of CoQ10 deficiency in the kidneys. Following CoQ10 supplementation, the level of CoQ10 increases in renal tissues, thus exerting antioxidant, anti-inflammatory, and anti-apoptotic effects. Among the 20 included studies, none of them reported CoQ10 status in blood, urinary, and renal tissue, before and after CoQ10 treatment. Thus, it is difficult to determine whether the protective effects elicited by CoQ10 supplementation in AKI are induced by the elevation of renal CoQ10 status. As a result, further clinical and experimental studies are warranted to better illustrate this phenomenon.

### Limitations and perspectives

To the best of our knowledge, this is the first collaborative review to summarize all the evidence of the protective properties of CoQ10 against AKI. CoQ10 is a commonly used compound and is well tolerated with few adverse effects, making it an attractive potential therapy for AKI. Nevertheless, some potential shortcomings deserve attention. First, only two clinical trials have reported the clinical implications of CoQ10 treatment in AKI; the remaining included studies were either in vivo or in vitro experiments. Thus, further clinical trials, and randomized, placebo-controlled double-blinded trials in particular, are still warranted to confirm the clinical significance of CoQ10 in AKI. Second, despite the use of various AKI animal models, the molecular mechanisms of CoQ10 in treating AKI have not been fully understood and should be investigated in future studies.

## Conclusion

The current review highlights the protective effects of CoQ10 in treating multiple types of AKI, including but not limited to AKI induced by drugs, ESWL, sepsis, contrast media, and ischemia–reperfusion injury. The renal protective roles of CoQ10 in AKI might be mainly due to its potent antioxidant, anti-apoptosis, and anti-inflammation properties. The underlying mechanisms for CoQ10 might be attributed to the regulation of multiple affected proteins (e.g., *caspase-3, p53*, and *PON1*) and signaling cascades (e.g., *Nrf2/HO-1* pathway). Upon further confirmation of the renal protective effects by extensive in-depth studies, CoQ10 administration may be a potential strategy for the treatment of AKI in clinical practice.

## Data Availability

The datasets used and/or analyzed during the current study are available from the corresponding author on reasonable request.

## Abbreviations

CoQ10: Coenzyme Q10
AKI: Acute kidney injury
iNOS: Inducible nitric oxide synthase
NF-κB: Nuclear factor-κB
PON1: Paraoxonase 1
HO-1: Heme oxygenase 1
ESWL: Extracorporeal shock wave lithotripsy
EGCG: Epigallocatechin gallate
MnSOD: Manganese superoxide dismutase
Nrf2: Nuclear factor erythroid 2-related factor 2
L-NAME: L-arginine analog
BUN: Blood urea nitrogen
SCR: Serum creatinine
ROS: Reactive oxygen species
